# The impact of modern migrations on present-day multi-ethnic Argentina as recorded on the mitochondrial DNA genome

**DOI:** 10.1186/1471-2156-12-77

**Published:** 2011-08-30

**Authors:** María Laura Catelli, Vanesa Álvarez-Iglesias, Alberto Gómez-Carballa, Ana Mosquera-Miguel, Carola Romanini, Alicia Borosky, Jorge Amigo, Ángel Carracedo, Carlos Vullo, Antonio Salas

**Affiliations:** 1 Equipo Argentino de Antropología Forense, Independencia 644 - 5C, Edif. EME1, Córdoba, Argentina; 2 Unidade de Xenética, Instituto de Medicina Legal and Departamento de Anatomía Patolóxica e Ciencias Forenses, Calle San Francisco sn, Facultade de Medicina, Universidade de Santiago de Compostela, CIBERER, Santiago de Compostela, 15782, Galicia, Spain; 3 Laboratorio de Inmunogenética y Diagnóstico Molecular, Independencia 644 - 4, Edif EME1, Córdoba, Argentina

## Abstract

**Background:**

The genetic background of Argentineans is a mosaic of different continental ancestries. From colonial to present times, the genetic contribution of Europeans and sub-Saharan Africans has superposed to or replaced the indigenous genetic 'stratum'. A sample of 384 individuals representing different Argentinean provinces was collected and genotyped for the first and the second mitochondrial DNA (mtDNA) hypervariable regions, and selectively genotyped for mtDNA SNPs. This data was analyzed together with additional 440 profiles from rural and urban populations plus 304 from Native American Argentineans, all available from the literature. A worldwide database was used for phylogeographic inferences, inter-population comparisons, and admixture analysis. Samples identified as belonging to hg (hg) H2a5 were sequenced for the entire mtDNA genome.

**Results:**

Phylogenetic and admixture analyses indicate that only half of the Native American component in urban Argentineans might be attributed to the legacy of extinct ancestral Argentineans and that the Spanish genetic contribution is slightly higher than the Italian one. Entire H2a5 genomes linked these Argentinean mtDNAs to the Basque Country and improved the phylogeny of this Basque autochthonous clade. The fingerprint of African slaves in urban Argentinean mtDNAs was low and it can be phylogeographically attributed predominantly to western African. The European component is significantly more prevalent in the Buenos Aires province, the main gate of entrance for Atlantic immigration to Argentina, while the Native American component is larger in North and South Argentina. AMOVA, Principal Component Analysis and hgs/haplotype patterns in Argentina revealed an important level of genetic sub-structure in the country.

**Conclusions:**

Studies aimed to compare mtDNA frequency profiles from different Argentinean geographical regions (e.g., forensic and case-control studies) should take into account the important genetic heterogeneity of the country in order to prevent false positive claims of association in disease studies or inadequate evaluation of forensic evidence.

## Background

The inhabitation of the Americas took place with the passage of people from northeast Asia to North America, who then rapidly moved southwards along the continent [1-4]. The first human settlements in Argentina were found in the Patagonia and dated to ~13,000 years ago (y.a.) [5]. The colonial period (roughly 1550-1810) began with the arrival of Spanish conquerors, and their domination lasted until the independence wars. During the colonial era, the Spaniards entered Argentina from Peru and Bolivia mainly through the northern 'Camino Real' and by the Río de la Plata, and they established a permanent colony on the site of what would later become Buenos Aires. Río de la Plata was also one of the main gates of entrance for other trans-Atlantic immigrants, such as African slaves. Indigenous people were under the domination of Spanish colonizers and many of these groups were exterminated or progressively admixed with the colonizers. Only natives inhabiting the mountainous north-western and southern Argentina survived the repression. At the end of the 19^th ^century, the Native populations were exterminated in the central region and upper Patagonia. The Argentinean National Constitution of 1853 promoted immigration from Europe, and the country received large waves of European immigrants, predominantly Italians (e.g., from South Italy) and Spanish (e.g., Galicia in northwest Spain [6]). In about 100 years, the census of Argentina increased by one order of magnitude to about 20 million people in 1960. Internal demographic movements were also important in Argentina during the industrialization period (1930-1950). Thus, waves of Native Americans moved from northern Native Argentinean enclaves to the largest cities of the country. In the seventies, massive numbers of immigrants would also arrive to the main cities coming from bordering countries (Bolivia, Paraguay, Uruguay, Chile, and Peru) [7-9].

Argentina is a melting pot of people with different continental ancestries but a majority of the citizens are descendents of colonial-era settlers and of the late19^th ^and early20^th ^century European immigrants. The official census in Argentina, INDEC (Instituto Nacional de Estadística y Censos; http://www.indec.gov.ar/), indicates that the country is populated by more than 40 million people, of which only about 600,000 (~1.7%) considered themselves as belonging to or descending from indigenous groups. About 30 officially recognized indigenous populations survived the colonial and post-colonial period up to the present and nowadays there are more than 25 Native speaking live languages [10]. The most important ones in terms of population size are the Mapuches in the South, and the Collas (also spelled Kollas), Tobas, Wichí and Guaraní in the North.

It is difficult to determine the real impact of the different demographic changes occurred in Argentina during the last few centuries. From a genetic point of view, one could indirectly predict the impact of the different contributors by looking at the census; however, the census can be somehow misleading for several reasons. Thus, the INDEC indicates that the proportion of Italians arriving to Argentina in the 1980 and 1991 was ~47% and ~51% involving about 236,467 and 167,977 individuals, respectively; while the Spaniards were 41% and 38% involving about 202,523 and 124,667 individuals, respectively. However, historical sources [7-9] indicate that Spain contributed more significantly to the Argentinean pool in several periods of the last 150 years (Table 1). On the other hand, the 'masculinity index' (as the amount of male immigrants each 100 female immigrants [8]) was larger for Italians than for Spaniards [8], which would contribute e.g. to inflating the signal left by Spaniards on the mitochondrial DNA (mtDNA) of contemporary Argentineans.

**Table 1:** Distribution of immigrants to Argentina coming from Italy, Spain and neighboring countries (modified from [8])

Inter-census period	Population (millions)	Immigrants (%)	Italians (%)	Spaniards (%)	M_IT_/M_SP_*^1^	Neighboring countries (%)
1869-1895	1.8-4.0	12.1	50.7	20.2	0.97	10.5
1895-1914	4.0-7.9	25.4	35.7	41.2	1.07	7.5
1914-1947	7.9-15.8	29.9	25	26.2	1.6	17.2
1947-1960	15.8-20.0	15.3	35.8	17.2	1.5	28.9
1960-1970	20.0	13	5.4	8.0	-	76.1

The percentages in the census indicate the approximate contribution regarding to the total immigrants. *^1 ^Lattes and Sautu [8] defines the 'masculinity index' (here denote as M) as the amount of man immigrants per 100 woman immigrants. Here, we define M_IT_/M_SP _as the quotient of the M value in Italians divided by the M value in Spaniards.

The study of mtDNA data has been demonstrated to be very useful in unraveling the patterns of human worldwide migrations, in particular, those occurred in America [1-3,11-15]. Several studies have been devoted to the analysis of mtDNA in Argentinean populations. Ginther et al. [16] analyzed the first hypervariable region (HVS-I) in a sample of indigenous Mapuches (South); the study revealed the predominant Native American nature of this population. Cabana et al. [17] analyzed the HVS-I of individuals belonging to different ethnic groups from Gran Chaco (North), and focused on the historical events occurring in this northern Argentinean region. Álvarez-Iglesias et al. [18] showed a SNP-based methodological approach to allocate Native American mtDNAs into hgs [18]. A sample from Córdoba (Argentina) was also analyzed by Salas et al. [19]; a high proportion of the Native American component was observed in the mtDNA lineages (~41%) but not on the Y-chromosome (~2%). Martínez-Marignac et al. [20] analyzed a sample from the city of La Plata (Central Argentina); the results corroborated the hg distribution observed in previous studies. In a sample from Argentina, the results of Bobillo et al. [21] showed that Amerindian hgs were most frequent in North and South (60%) and decreased to less than 50% in Central. García and Demarchi [22] reported hg frequencies in nine villages from central Argentina, indicating that ~80% of the lineages belonged to native American hgs. In a congress report, Catelli et al. [23] presented broad hg frequencies of a subset of the sample used in the present study. Mitochondrial DNA sequences were also investigated in six Mbyá-Guaraní villages (northeastern) [24], being A2 and D1 the ones exhibiting the highest frequencies (~41% and ~36%, respectively). Most recently, Corach et al. [25] investigated the genetic admixture of unrelated male individuals from eight different provinces using different sets of markers; the results showed that different ancestry components were detectable in contemporary Argentineans, the amounts depending on the genetic system applied, exhibiting large inter-individual heterogeneity.

The present study has been motivated by the following reasons: (i) although several Argentinean populations have been analyzed to date, Argentina has not been analyzed from a global perspective (with the exception of the [25] study which however focus on a different sampling strategy, different methodology and aims), and several regions still remain uncharacterized, (ii) there is a need to explore the levels of population stratification within the Argentinean country since this could have important consequences in different biomedical studies, (iii) a comprehensive and comparative analysis of the mtDNA patterns observed in Native communities *versus *rural and urban population is still lacking, and (iv) while Native American lineages in Argentina have been analyzed with certain resolution, the provenience of the trans-Atlantic immigration has been poorly inferred from control region sequences.

## Methods

### DNA samples

**Figure 1:**
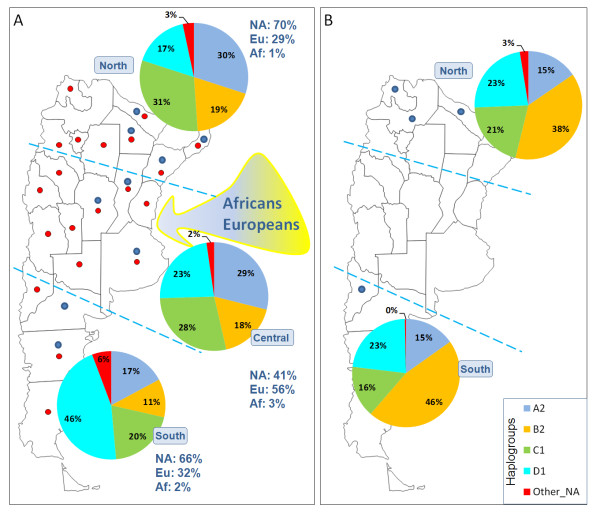
**Frequency patterns of the main hgs in Argentina in the admixed groups (A) versus the Native American communities (B).** NA: Native American component; Eu: European component; Af: sub-Saharan African component. Red dots indicate sampled locations as undertaken in other studies from the literature; blue dots indicate the sampled locations in the present study.

A total of 384 blood samples were collected from unrelated donors by the Equipo Argentino de Antropología Forense, and the Laboratorio de Inmunogenética y Diagnóstico Molecular de Córdoba representing different regions in Argentina (Figure 1). All the participants have permanent residence in Argentina. An undetermined proportion of them could descent from non-Argentinean parents or great-parents but this information was not recruited. One of the aims of the present study was to evaluate the proportion of Native American component that is autochthonous *versus *non-autochthonous in people that have permanent residence in the country. The analysis provides therefore a rough estimate of the amount of autochthonous lineages that are among present-day Argentineans. On the other hand, since we have carried out a meta-analysis of Argentinean mtDNA profiles adding to our set of lineages those collected from the literature, uncertainty exists concerning the characterization of many donors (see discussion below).

The geographic origin and sizes of the samples analyzed in the present study are summarized in Additional file 1: Table S1. Broad hg frequencies of a subset of these samples have been summarized in a previous congress report [23].

DNA was extracted using phenol-chloroform standard procedures. Written informed consent was obtained in Argentina from all the participants. In addition, an Institutional Ethical approval to carry out this study was obtained from the Equipo Argentino de Antropología Forense (EAAF) and the University of Santiago de Compostela.

### PCR, sequencing and minisequencing analysis

Samples were PCR amplified and sequenced for HVS-I and HVS-II regions as described previously [23]. In addition, all the profiles were contrasted with the phylogeny in order to detect potential artifacts e.g. [26]. In order to increase the phylogenetic resolution, most of the samples were genotyped for sets of diagnostic SNPs mainly located in the coding region (mtSNPs). For the samples belonging to R0 (European ancestry), a set of 71 mtSNPs were genotyped as described previously [27] whereas samples belonging to Native American hgs were additionally genotyped for 31 mtSNPs as described in [18]. The full set of results for the control region sequences and the mtSNPs are shown in Additional file 2: Table S2.

### Population database

A database of mtDNA profiles of rural and urban populations (referred to in this article as the admixed group/population) and indigenous Argentineans has been compiled from the literature. Together with the samples analyzed here, the Argentinean database contains 824 mtDNAs representing 24 different populations. The Native American groups were collected from (a) North Argentina (*n *= 265), and includes Coyas (*n *= 61) from the provinces of Jujuy and Salta [18], Pilagá (*n *= 38) and Toba (*n *= 24) from Gran Chaco (Formosa), Toba (*n *= 43) from Chaco (Formosa), and Wichí or Mataco (*n *= 99) from Gran Chaco [17], and (b) South Argentina, represented by 39 Mapuches [16]. The admixed populations were collected from: (a) North Argentina (*n *= 98), including Formosa (*n *= 19), Chaco (*n *= 5), Misiones (*n *= 48) and Corrientes (*n *= 26) [21]; (b) Central Argentina (*n *= 295) from Santa Fe (*n *= 6) and Buenos Aires (*n *= 187) [21] and Córdoba (*n *= 102) [19]; and (c) South Argentina (*n *= 47) from Río Negro (*n *= 46) and Chubut (*n *= 1) [21].

In addition, data from ancient DNA studies [28] or other studies aimed to target specific mtDNA lineages (such as [1]) were also used for database searching.

A database of European (Italian and Spanish) and other Argentinean neighboring populations (including Uruguay, Paraguay, Bolivia and Chile) were additionally used for the admixture analysis. Details on the samples used in this study are provided in Additional file 1: Table S1.

### Admixture analysis

**Figure 2:**
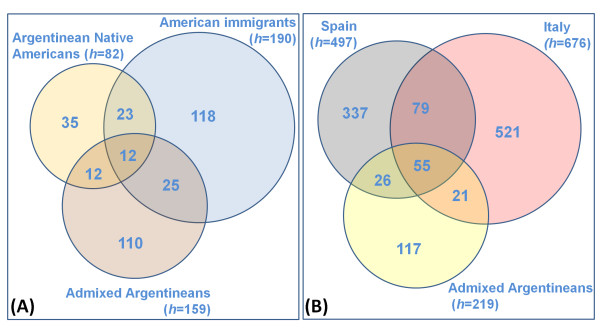
**The share of identical haplotypes (H) between: (i) the Native American component of the admixed Argentinean populations (salmon-pink) versus the Argentinean Native Americans (yellow) and American immigrants (light blue) (Paraguay, Bolivia, Uruguay and Chile) (Figure 2A); and (ii) the European component of the admixed Argentinean populations (yellow) and a database of the Spanish (gray) and Italians (pink) (Figure 2B)**.

Here, we are interested in separately analyzing the origin of the European and the Native American component of urban Argentineans. It was known from the Argentinean census that Spain and Italy were the two main countries in supplying European immigrants to Argentina. In modern times, Argentina has been also the destination of thousands of immigrants coming from neighboring countries that have a predominant Native American component. A premise of admixture analysis is that the source populations considered in the model are genetically different. Figure 2 indicates this feature by way of exploring the number of sharing haplotypes between the population groups involved in the admixture analysis. Differences between Italy and Spain are small and cannot be detected when looking at statistical tests of population differentiation (yielding non-significant statistical differences; data not shown) or examining genetic distances (F_ST _= 0.0022); an issue that could be improved in the future if adding more molecular information to the statistical model (e.g. entire genomes and larger sample sizes). Although F_ST _is not informative at indicating differences between Spain and Italy, and given the fact that one of the admixed analysis carried out in the present study (see below) relies on haplotype sharing, we have carried out a simulation analysis in order to test if the two populations are sufficiently different in terms of haplotype sharing in order to support the results yielded by the admixture analysis. We performed a simulation that consists of (i) randomly distributing in 10,000 iterations the total number of individuals (from Spain and Italy jointly considered) in two groups (with samples sizes as in the original samples), (ii) compute the proportion of shared haplotypes each time, and (iii) reconstruct the distribution of this statistics under the null hypothesis of no differentiation. Clearly, the observed haplotype sharing is significantly smaller than 5^th ^percentile of this distribution (see Additional file 3: Figure S1). This allowed to conclude that haplotype sharing contains enough information to discriminate Spain and Italy and therefore to compute admixture proportions of Argentineans from Europe.

The first admixture model was undertaken as described by Salas et al. [29]; see also [30]. Since this model is based on hg frequencies, it was only applied to infer the contribution of the European countries to the population of Argentina. This is because the Native American component was too homogeneous and the phylogenetic hg resolution was too low (at the control region level) to yield meaningful results.

The second admixed model was applied as described previously [14], but with an extension of the original model that is detailed below. The probability of origin of each of the sub-continental region can be computed as $po_{s}=\frac{1}{n}\sum_{i=1}^{n}k_{i}\frac{p_{is}}{p_{iC}}$, where *n *is the number of Argentinean sequences with matches (≥ 1) in the whole database; *k_i_*, the number of times the sequence *i *is found in Argentina; *p_is_*, the frequency of the sequence *i *in each regional datasets (e.g., Spain and Italy); and *p_ic_*, the frequency of the sequence *i *in the whole database. The same analysis was carried out independently considering *n *to be the number of Argentinean sequences that have cero, one or two mutational differences from the sequences contained in the database. We will refer to *P_0_, P*_1_, and *P_2_*, for the admixture components of sequences that match perfectly, differ by one mutational step, or two, respectively. In order to account for different sample sizes in the source populations, admixed components (and their 95% C.I.) were built by way of bootstrapping, taken 1000 re-samples of the source populations of size 300 each (other sample sizes yielded consistent results; data not shown).

### Statistical analysis

DnaSP v.5 software [31] was used for the computation of haplotype (*H*) and nucleotide (*π*) diversities, and mean number of pairwise differences (*M*). AMOVA (Analysis of Molecular Variance) and the significance of the covariance components associated with different levels of genetic structure were tested on haplotypes and haplogroup frequencies applying a non-parametric permutation procedure. The latter analyses and population pairwise F_ST _values, between/within population average nucleotide pairwise differences, and Nei's inter-population distances, were computed using Arlequin 3.5.1.2 [32]. Diversity indices, phylogeographic inferences and inter-population comparisons were carried out using the sequence range 16090 to 16365, since this is the common segment reported in the literature. Problematic variation located around 16189 usually associated to length heteroplasmy, e.g., 16182C or 16183C, was ignored. Principal Component Analysis (PCA) was carried out on population hg frequencies using R http://www.r-project.org/. AMOVA and PCA were performed on Argentinean samples of sample sizes ≥ 20 (see Additional file 1: Table S1).

Fisher's exact test and Pearson's chi-square test were undertaken using the R package http://www.r-project.org/, a significant value of α = 0.05 was considered.

Finally, estimation of the time to the most recent common ancestor (TMRCA) and SDs of hg H2a5 were carried out according to Saillard et al. [33]and using an evolutionary rate estimate for the entire mtDNA molecule as reported by Soares et al. [34].

## Results

### Summary statistics in Argentinean mtDNAs

Summary statistics were computed for admixed Argentineans, Native Americans, and the whole Argentinean sample (Table 2). The analysis was also carried out separately for the the Native American and the European components (Table 2). The Native American component of the admixed populations has higher diversity values than the one of the indigenous groups (Table 2) for all the indices computed. Within the admixed groups, there is more sequence diversity in Central and South while nucleotide diversity is higher in Argentina.

**Table 2:** Diversity indices in the Argentinean population groups

	*N* [1]	*H* [1]	*H/n* [1]	*D* [1]	*Π* [1]	*M* [1]	*N* [2]	*H* [2]	*H/n* [2]	*D* [2]	*Π* [2]	*M* [2]	*N* [3]	*H* [3]	*H/n* [3]	*D* [3]	*Π* [3]	*M* [3]
Urban populations																		
North	37	28	0.76	0.980 (0.012)	0.0155 (0.0015)	4.9	90	46	0.51	0.958 (0.010)	0.0199 (0.0008)	6.3	129	76	0.59	0.978 (0.005)	0.0208 (0.0007)	6.6
Central	358	195	0.54	0.978 (0.004)	0.0131 (0.0005)	4.1	263	118	0.45	0.965 (0.005)	0.0196 (0.0004)	6.2	642	329	0.51	0.987 (0.002)	0.0187 (0.0004)	5.9
South	17	14	0.82	0.971 (0.001)	0.0134 (0.0024)	4.2	35	27	0.77	0.971 (0.018)	0.0187 (0.0013)	5.9	53	42	0.79	0.985 (0.009)	0.0192 (0.0010)	6.1
All	412	216	0.52	0.978 (0.004)	0.0133 (0.0004)	4.2	388	158	0.41	0.967 (0.004)	0.0197 (0.0003)	6.2	824	392	0.48	0.987 (0.001)	0.0192 (0.0003)	6.0
Native Americans																		
North	-	-	-	-	-	-	265	72	0.27	0.940 (0.008)	0.0181 (0.0005)	5.7	265	72	0.27	0.940 (0.008)	0.0181 (0.0005)	5.7
South	-	-	-	-	-	-	39	13	0.33	0.908 (0.020)	0.0171 (0.0006)	5.4	39	13	0.33	0.908 (0.020)	0.0171 (0.0006)	5.4
All	-	-	-	-	-	-	304	82	0.27	0.950 (0.006)	0.0181 (0.0004)	5.7	304	82	0.27	0.950 (0.006)	0.0181 (0.0004)	5.7
All Argentineans																		
All	-	-	-	-	-	-	1128	449	0.40	0.984 (0.001)	0.0192 (0.0003)	*id*	1128	449	0.40	0.984 (0.001)	0.0192 (0.0003)	6.0

Standard deviations are given in round brackets.

*n *= sample size, *H *= number of different haplotypes, *D *= sequence diversity, *π *= nucleotide diversity, *M *= average number of pairwise differences. Numbers in square brackets (above) indicate: [1]: haplotypes of European ancestry,[2]: haplotypes of Native American ancestry, [3]: all the haplotypes together

The diversity of European lineages in the admixed group is higher in the North (Table 2). As expected, the European component is more diverse than the Native American one (Table 2) for the haplotype diversity, corresponding with their demographic histories, which is about four times older for the Europeans than for the Native Americans with the latter suffering strong bottlenecks at the time of entrance through the Bering Strait [1-4]; nucleotide diversity shows the opposite pattern which in this case most likely mirrors the low resolution of the HVS-I in a high proportion of European lineages (e.g. macro-hgs R0). Finally, admixed groups are genetically more diverse than the Native American ones (Table 2).

### Phylogeography of mtDNA lineages in Argentina

The Native American component observed in the urban populations was 66%, 41%, and 70% in South, Central, and North, respectively (Figure 1) and it was virtually 100% in most Native American groups. The distribution of Native American hgs was substantially different in the main Argentinean regions especially when looking at urban populations (Figure 1A); for instance, hg A2 constitutes 30% in North admixed populations but only 17% in South admixed populations (Figure 1A) (Pearson's χ^2 ^test; un-adjusted *P*-value = 0.00561). Moreover, the percentages of the different Native American hgs significantly differ when comparing admixed with native populations (Figure 1A vs Figure 1B), even when comparing samples from the same geographical location; thus, for example, when considering only the Native American component of the urban populations, hg B2 is 19% in North admixed populations *versus *38% in North Natives (Pearson's χ^2 ^test; un-adjusted *P*-value = 0.01808), or hgs B2 and D1 have frequencies of 11% *versus *46% (hg B2; Pearson's χ^2 ^test; un-adjusted *P*-value < 0.0000) and 46% *versus *23% (hg D1; Pearson's χ^2 ^test; un-adjusted *P*-value = 0.00334) in South admixed populations *versus *South Natives.

The lower prevalence of Native American hgs observed in Central Argentina coincides with the high proportion of European lineages in this region, mirroring the fact that this was the main European settlement area in the country; e.g. the European component is significantly more predominant in Central (56%) than in North (29%; Pearson's χ^2 ^test; un-adjusted *P*-value < 0.00901).

African slaves were brought to Argentina by Europeans during the period of the Atlantic slave trade [30,35,36] and they entered the country following the main entrance provided by the Río de la Plata, but the impact of this process in the mtDNA pool of Argentina was much lower than in other American regions [11,14,37,38]. Sub-Saharan lineages represent only 1-3% of the total mtDNA component observed in Argentina.. The most prevalent sub-Saharan HVS-I mtDNAs in Argentina are: (i) the L2c2 profile C16223T C16264T C16278T T16311C, which also appears in Brazil [38,39] and other American locations [40]; exact matches of this mtDNA profile were found in Gabon [41], Cabinda [42], Mozambique [43] and some other South African locations; and (ii) the L3f1a mtDNA G16129A T16209C C16223T C16292T C16295T T16311C that also appeared in Brazil [39,44] and in US 'African Americans' [40,45]; this hg has a likely origin in East Africa [43] but probably arrived in America via West-Central Africa [41] or Southwest Africa [42]; see also [36]. Other typical North African profiles belonging to hg U6 reached Argentina via Portugal or Madeira (such as T16172C C16174T C16188T A16219G T16311C; hg U6b) [46,47], Canary islands (G16129A C16169T T16172C T16189C [48]) or directly from Morocco (T16172C A16183C T16189C A16219G C16239T C16278T T16362C [49]). Only two Argentinean mtDNAs belong to hg M1, the hg that is prevalent in the Middle East and East Africa and with a wide distribution in several African regions. For instance, matches for G16129A T16189C C16223T T16249C T16311C T16359C were observed in the Chad Basin [50], Ethiopia [51] and Egypt [52] while profile G16129A T16189C T16249C T16311C is present only in the Arabs in Chad [50] and outside Africa in Spain [53,54].

Finally, it is also interesting to note that haplotype frequencies vary substantially between populations (Additional file 4: Figure S2). For instance, Native American groups have several haplotypes at high frequencies (probably due to historical bottlenecks).

### Admixture analysis and the mtDNA indigenous legacy in present-day Argentina

Admixture analysis, as carried out here, considers two potential source populations: (i) the Native American component of the available indigenous Argentinean populations, and (ii) the Native American component of neighboring countries as a proxy for the Native American component that has been introduced into Argentina through recent immigration. The model of admixture (Table 3) indicates that about half of the Native American component in the urban populations most likely comes from immigration arriving from neighboring countries, while the rest most likely corresponds with the indigenous inhabitants living in those regions before European colonization or arriving from rural Argentinean Native American enclaves. The data is roughly consistent when executing admixture analysis either looking at full HVS-I matches (*P*_0_) or considering one or two mutational steps (*P*_1 _*and P*_2_).

**Table 3:** Admixture proportions, *P*_0_, *P*_1_, *P*_2 _(95% C.I. in brackets) of admixed Argentineans referring to their Native American (mainly hgs A2, B2, C1 and D1) and European components according to the main source populations

Urban Argentinean Populations (*n *= 800)	Argentinean Native Americans (*n *= 303; *h *= 82)	Argentinean Native Americans (*n *= 303; *h *= 82)	Argentinean Native Americans (*n *= 303; *h *= 82)
Native American Component (*n *= 388)	*HS_0 _*= 25 (0.30)	*HS_1 _*= 96 (1.17)	*HS_2 _*= 141 (1.72)
Native American Component (*n *= 388)	*P*_0 _= 0.50 (0.03)	*P*_1 _= 0.46 (0.03)	*P*_2 _= 0.42 (0.03)
	**American immigrants** **(*n *= 488; *h *= 190)**	**American immigrants** **(*n *= 488; *h *= 190)**	**American immigrants** **(*n *= 488; *h *= 190)**
Native American Component (*n *= 388)	*HS_0 _*= 38 (0.20)	*HS_1 _*= 108 (0.56)	*HS_2 _*= 144 (0.76)
Native American Component (*n *= 388)	*P*_0 _= 0.50 (0.03)	*P*_1 _= 0.54 (0.03)	*P*_2 _= 0.58 (0.03)
	**Spain** **(*n *= 1467; *h *= 497)**	**Spain** **(*n *= 1467; *h *= 497)**	**Spain** **(*n *= 1467; *h *= 497)**
European component (*n *= 412)	*HS_0 _*= 81 (0.16)	*HS_1 _*= 159 (0.32)	*HS_2 _*= 174 (0.35)
European component (*n *= 412)	*P*_0 _= 0.55 (0.5499-0.5564)	*P*_1 _= 0.49 (0.4896-0.4961)	*P*_2 _= 0.50 (0.5009-0.5074)
	**Italy** **(*n *= 1667; *h *= 676)**	**Italy** **(*n *= 1667; *h *= 676)**	**Italy** **(*n *= 1667; *h *= 676)**
European component (*n *= 412)	*HS_0 _*= 76 (0.11)	*HS_1 _*= 177 (0.26)	*HS_2 _*= 185 (0.27)
European component (*n *= 412)	*P*_0 _= 0.45 (0.4436-0.4501)	*P*_1 _= 0.50 (0.5039-0.5104)	*P*_2 _= 0.49 (0.4926-0.4991)

Shared haplotypes between groups are also indicated; *HS_0_, HS_1 _*and *HS_2 _*refer to the number of shared haplotypes differing 0, 1 or 2 variants between the two main components of the urban mtDNAs (Native American and European) and the number of haplotypes (*h*) in the corresponding source populations. Note that these values can be > 1 because the same haplotype in the source population can count more than once for *HS_1 _*and *HS_2_*. The amount *n *indicates sample size. *P*_0_, *P*_1_, and *P*_2_, are the admixture components referring to sequences that match perfectly, differ by one mutational step, or two, respectively, in the database; standard deviations are in round brackets.

### Characterizing the most likely origin of the European component in present-day Argentina

The models employed here considers only the two main historical contributors to the European immigration in Argentina, namely Italy and Spain (representing > 80% of immigrants coming from Europe in the last 150 years; Table 1).

The mathematical admixed model based on hg frequencies indicates that Italy most likely contributed 33% (95% SD: 9.2) of the European mtDNA hgs to the Argentinean genome versus 67% (95% SD: 8.1) from Spain. The admixed model based on haplotype sharing yielded slightly different but quite consistent results, roughly indicating that Spain and Italy contributed almost similarly to the European component in Argentina (Table 3), although there are slight differences when considering perfect haplotype matches (*P_0_*; indicating ~55% contribution from Spain) *versus *considering one or two mutational step differences between HVS-I profiles (*P_1 _*and *P_2_*, indicating about equal contribution from Spain and Italy). The haplotype shared between Argentina and Europe seems to favor the hypothesis that the Spanish legacy in Argentina is slightly larger than the one from Italy (Table 3), either when looking at perfect haplotype matches (*HS_0_*) or one (*HS_1_*) or two (*HS_2_*) mutational step differences.

### AMOVA analysis of Argentinean populations

When applying AMOVA on haplotypes, variance within populations accounts for ~84% of the total variance (Table 4) Grouping populations by geographic region or by Native American *versus *Admixed populations add little to the proportion of variance among groups (~1; Table 4); probably indicating that the HVS-I alone does not provide enough molecular information for the computation of F_ST _based on molecular distances (pairwise differences). However, when applying AMOVA on haplogroup frequencies, among groups variance, by geography or by admixed vs Native groups, increases substantially to ~4 and ~6%, respectively. The figures are however not very high given that about half of the component of the admixed populations is Native American.

**Table 4:** AMOVA of Argentinean populations

	Within Populations	Among Groups	Among Pop./Among Pop. within groups
Pairwise differences			
All populations	83.72	-	16.28
North/Central/South	83.43	1.04	15.53
Native/Admixed	83.67	0.10	16.22
Haplogroup Freq.			
All populations	75.48	-	24.52
North/Central/South	74.63	3.39	21.98
Native/Admixed	73.18	6.09	20.73

Values indicate the distribution of the variance components according to the different hierarchical population levels; all of them are statistically significant (Significant tests: 20,022 permutations; adjusted *P*-value < 0.0000)

Additional file 5: Figure S3A displays population pairwise F_ST _values, indicating that the highest figures occur in comparisons involving Native American populations. Nei's genetic distances are in good agreement with pairwise F_ST _matrix values (Additional file 5: Figure S3B). Population structure is also reveled when observing that values of the average number of nucleotide differences between are higher than those for within population comparisons (Additional file 5: Figure S3B).

### Principal component analysis of Argentinean populations

**Figure 3:**
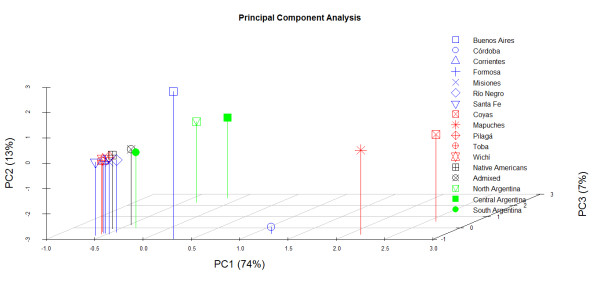
**Principal Component Analysis of Argentinean populations. PC1, PC2 and PC3 stand for principal component one, two and three, respectively**.

PCA was carried out on hgs frequencies for Argentinean samples with sizes > 20 (Figure 3). PC1 accounts for 74% of the variation; it clearly separates Mapuches and Coyas to one side of the plot, from an amalgam of other population samples in the opposite side; Buenos Aires and Córdoba occupy an intermediate position. PC2 (13%) is clear at showing an important separation between the two admixed populations of Buenos Aires and Córdoba; the rest of the populations are located in between. The most important feature of PC3 (7%) is that it separates populations by geographic regions, with South being more distant from Central and North (Argentina). It is important to highlight that the merged groups of admixed and Native American populations are located very proximal in the plot (Figure 3) in agreement with AMOVA results.

### Complete H2a5 genomes

**Figure 4:**
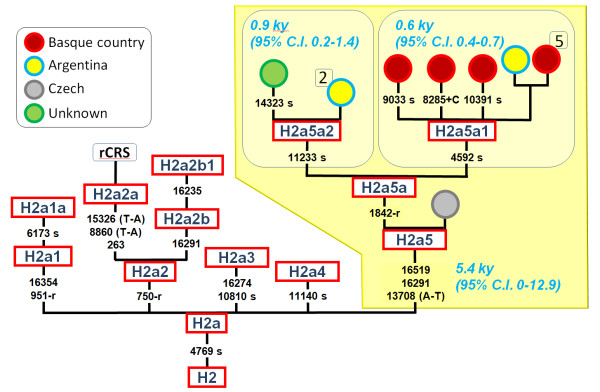
**Phylogeny of hg H2a5 based on complete genome sequences**.

Three entire genomes belonging to the recently described lineage, H2a5, have been completely sequenced. One of the entire Argentinean genomes belongs to the H2a5a1 branch (previously H2a5 [27]) defined by the transition T4592C (Figure 4). This clade has only been observed in the Basque country where it is supposed to be autochthonous [27]. The other two entire H2a5 genomes analyzed from Argentina are identical and belong to a new branch (defined by a synonymous transition at position T11233C), H2a5a2. The only known member belonging to this clade was observed by Achilli et al. [55]. The geographical location of its donor is unknown although his surnames (A. Achilli, personal communication) suggest a Galician origin (a region located in the westernmost corner of the Cantabrian region [6]); one of the main Spanish source populations to Argentina. The age of H2a5 is approximately 5.4 thousand years (kya) (95% C.I.: 0-12.9 kya) but the Basque autochthonous sub-clade H2a5a1 is much younger (~0.6 kys; 95% C.I.: 0.4-0.7 kya).

Finally, there is another entire genome sharing the same features as H2a5. It does not carry private mutations, lacks transition A1842G and was observed outside the Iberian Peninsula in the Czech Republic [56].

## Discussion

Admixed Argentineans have an important Native American background. Admixture models indicated that about half of this Native American component could be non-autochthonous. The exact figures are only tentative given a main limitation of the present study, namely, we did not collect bio-geographic information for most of the donors of our samples, and this is information was not available for most of the data collected from the literature; therefore, some donors could be in reality Native American immigrants (or descents form parents) from neighboring countries. Given the results of admixture analysis, one could tentatively hypothesize an important demographic influence coming from neighboring countries that have a predominantly Native American background and where massive immigrations to Argentina have come from in recent times (such as Paraguay, Peru and Bolivia). There are several other pieces of evidence that would further support this hypothesis. Firstly, the Native American component in the urban admixed populations differs very significantly from the Native American component of the indigenous populations from North and South Argentina (Figure 1). A simple process of (recent) admixture of Europeans with indigenous peoples would tend to keep the same hg frequencies in admixed and indigenous people, which is not the case here. Secondly, several diversity indices are significantly higher in the Native American component of admixed Argentineans than in the indigenous groups (see above and Table 2). This could be easily explained if one assumes that the Native American component in the admixed populations has being continuously enriched with the arrival of a different Native American component coming from recent neighboring immigrants together with migrants arriving from rural Argentinean regions with large Native American components (from northeast and northwest Argentina). On the contrary the indigenous groups would tend to reduce its genetic diversity with time due to drift (smaller effective population size) and isolation from Europeans and other immigrants. The data therefore indicates that the Native American component observed in the urban groups only partially mirrors the populations that inhabited the regions in colonial times. An important proportion of the autochthonous Argentinean Native American component could have arrived to rural and urban cities in modern times. For instance, after the economical crisis suffered in the country in the 1930's, waves of people from rural areas with high Native American component moved to industrialized cities [7-9].

From the different analyses carried out, the contribution of Spain in the present Argentineans seems to be slightly higher than that of Italy, although the estimates vary significantly depending on the admixture model. The results agree quite well with the historical records (Table 1). Thus, until 1850 almost all immigrants came from Spain. From 1850 onwards, thousand of Spaniards and Italians left their countries with final destiny in Argentina; but Spaniards were generally more prevalent than Italians [7-9]. Moreover, 'Spanish ancestry' could have enriched the Argentinean European component through immigrants coming from neighboring countries, where Spaniards contributed significantly more than Italians (Uruguay, Chile, etc).

Some caveats should be added concerning admixture analysis. Computations are based on a meta-analysis by way of collecting samples that did not necessarily follow the same sampling criteria. Thus, for instance, samples from Argentina were collected in different forensic, anthropological or clinical laboratories, using different sampling criteria; a meta-analysis could contribute in the direction of balancing different sampling strategies or the opposite in case e.g. of some sample being much larger than others. Moreover, it is well-known from the census that some regions in some (European/American) countries contributed more than others to Argentina; but it is not possible to determine how the different regions should be represented in the source meta-populations; a reasonable solution seems to merge all the available data from each country without any *a priori *regarding sampling origin or institution involved (as done in the present study).

Admixture analysis as carried out in the present study only provides a view of the female historical and contemporary demography (as inferred from the mtDNA); there are however indications showing that the ancestral proportions inferred from other markers are different [19,25], indicating for instance a sex bias in the contribution coming from the different source populations (at least from Europe).

It is also interesting to note that the genetic diversity of European lineages in the admixed groups is higher in the North than in the other regions, independently to the fact that the proportion of European in higher in Central Argentina. This is consistent with the historical documentation indicating that the 'Camino Real' to Potosí (Bolivia) and Lima (Peru) was by far the most important trade route during colonial times. Thus, Río de la Plata was the main gate for European immigrants into Argentina in modern times, but contributing less mtDNA diversity than the northern 'Camino Real'.

The impact of the African slave trade on present day Argentineans seems minimal compared to other South American locations (e.g., Brazil and Colombia), and comes most likely from West-central Africa, but also from Angola and Mozambique (see [30]).

## Conclusion

The issue of population stratification in Argentina has stimulated an intense debate concerning the use of autosomal markers in forensic casework and paternity tests. While some hold a position that stratification is an issue of little interest in forensic databases [57], others claim a more important role in both forensic and clinical genetics [19,58-61,19,21]. The present study certainly indicates the existence of a clear-cut sub-structure in the country; this is shown by the differences observed in hg distributions, AMOVA analysis, population differentiation tests, statistical hypothesis testing on hg frequencies, and PCA. Population stratification could have obvious implications in different biomedical applications in Argentina. This would not only be in forensic genetics (where inter-population haplotype differences can have important consequences for the weight of the evidence) but also in other population-based studies (e.g., case-control studies) dealing with the analysis of the potential role of mtDNA variants in common diseases, where false positives are unfortunately higher than desirable [62-64]. By extrapolation, and given the important ancestral components and regional differences observed in the mtDNA variation, stratification should also be a matter of interest when using autosomal SNPs. The forensic field should not ignore forensic stratification in their routine casework [60,65], especially if one takes into account that local databases do not exist in Argentina and that most of the forensic casework is carried out in the largest cities under the risk of using a single database for cases arriving from any province in the country.

## Competing interests

The authors declare that they have no competing interests.

## Authors' contributions

MLC, VAI, AGC, AMM, CR, AB and CV carried out the genotyping of the samples used in the present study. AS carried out the meta-analysis and statistical analysis, and drafted the manuscript, and JA performed the simulation analysis. CV, AC, and AS contributed materials and reagents. All authors approved the final version of the manuscript.

## Supplementary Material

Additional file 1 **Table S1**. List of samples analyzed in the present study and collected from the literature (American neighboring countries and European ones) used for admixture analysis. Click here for file

Additional file 2 **Table S2**. Haplotype and mtSNP profiles of the Argentinean samples analyzed in the present study. Click here for file

Additional file 3 **Figure S1**. Simulation aimed to demonstrate that Italy and Spain are sufficiently different in terms of haplotype sharing, therefore, supporting the results of admixture analysis. First, two databases were considered jointly, the Spanish (n = 1467) and the Italian database (n = 1667) (see Additional file 1: Table S1, and text for more information on the databases). From this global database (n = 3134), two samples of sizes 1467 and 1667 each were taken at random without replacement 10,000 times. The distribution represents the number of identical shared haplotypes (horizontal axis) and their counts (vertical axis) between the 10,000 pairs of random samples. The red line indicates the observed number of haplotypes shared between the Italian and the Spanish database (n = 134; see also Figure 2). Click here for file

Additional file 4 **Figure S2**. Patterns of haplotype frequencies in Argentinean population samples. Only those samples of sizes > 20 individuals were considered. Click here for file

Additional file 5 **Figure S3**. Pairwise F_ST _values (A), and average number of pairwise differences within and between populations and Nei's distances (B). Click here for file
