# Supplementary figures and images for: The impact of modern migrations on present-day multi-ethnic Argentina as recorded on the mitochondrial DNA genome
Source: BMC Genet. 2011 Aug 30;12:77. doi: 10.1186/1471-2156-12-77 (PMC3176197; doi:10.1186/1471-2156-12-77)

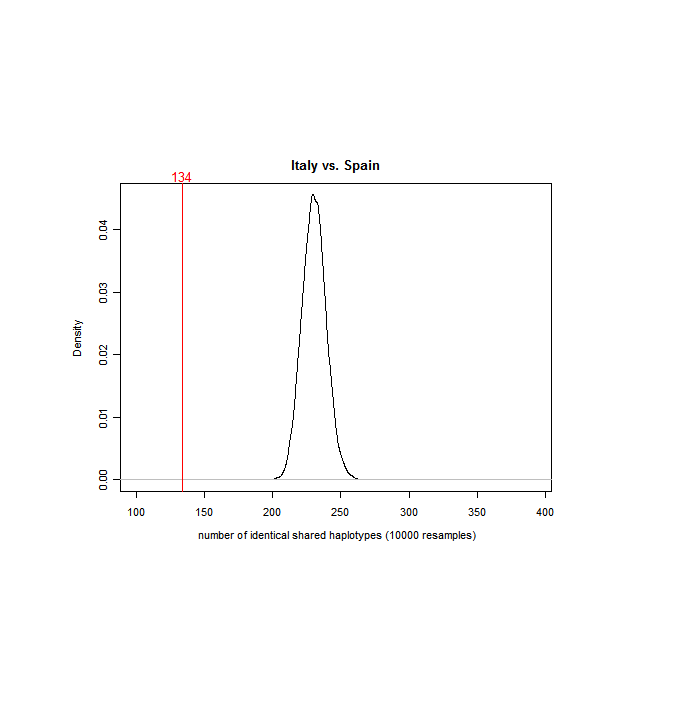

Supplement: Additional file 3 — Figure S1. Simulation aimed to demonstrate that Italy and Spain are sufficiently different in terms of haplotype sharing, therefore, supporting the results of admixture analysis. First, two databases were considered jointly, the Spanish (n = 1467) and the Italian database (n = 1667) (see Additional file 1: Table S1, and text for more information on the databases). From this global database (n = 3134), two samples of sizes 1467 and 1667 each were taken at random without replacement 10,000 times. The distribution represents the number of identical shared haplotypes (horizontal axis) and their counts (vertical axis) between the 10,000 pairs of random samples. The red line indicates the observed number of haplotypes shared between the Italian and the Spanish database (n = 134; see also Figure 2). [file 1471-2156-12-77-S3.TIFF]

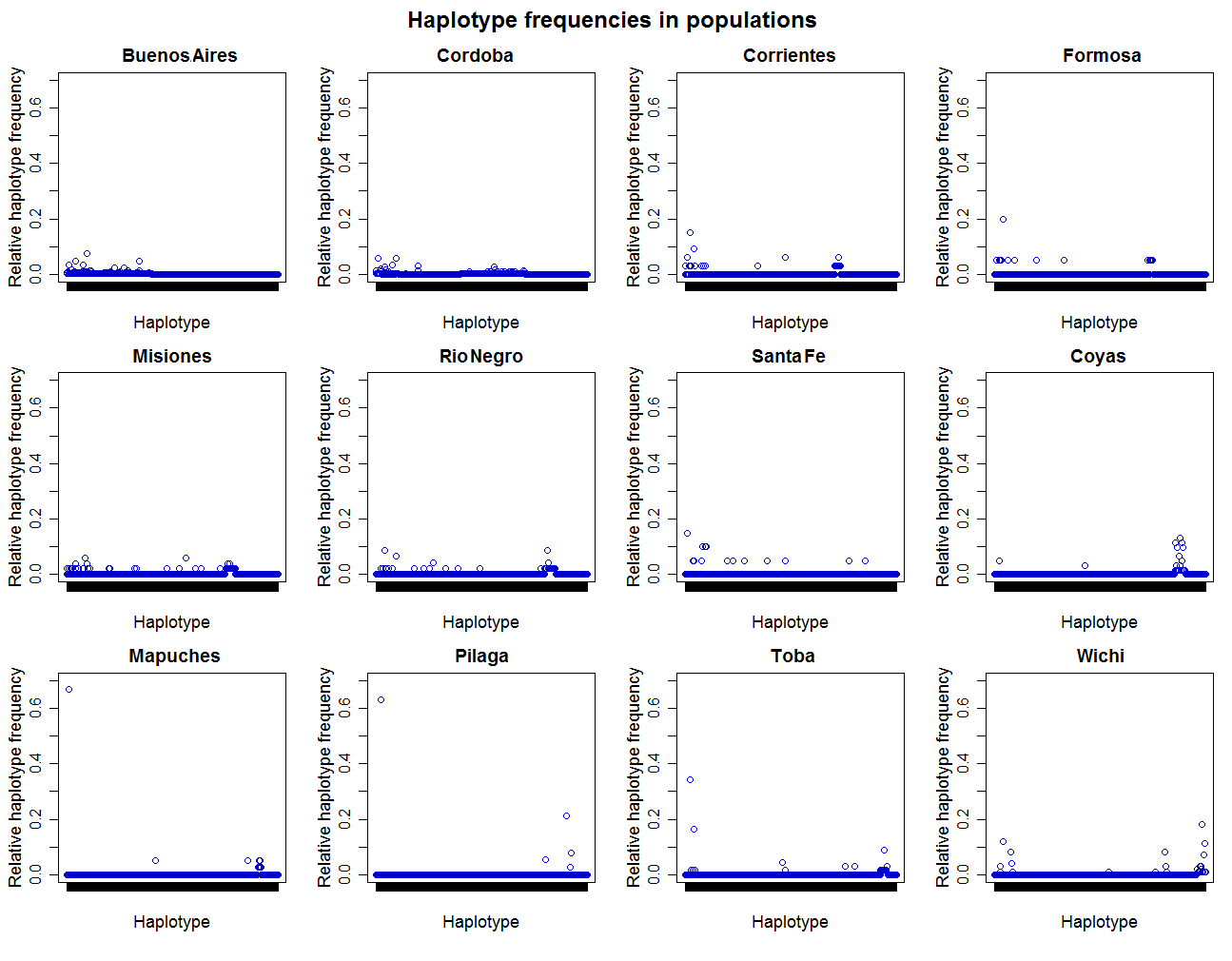

Supplement: Additional file 4 — Figure S2. Patterns of haplotype frequencies in Argentinean population samples. Only those samples of sizes > 20 individuals were considered. [file 1471-2156-12-77-S4.TIFF]

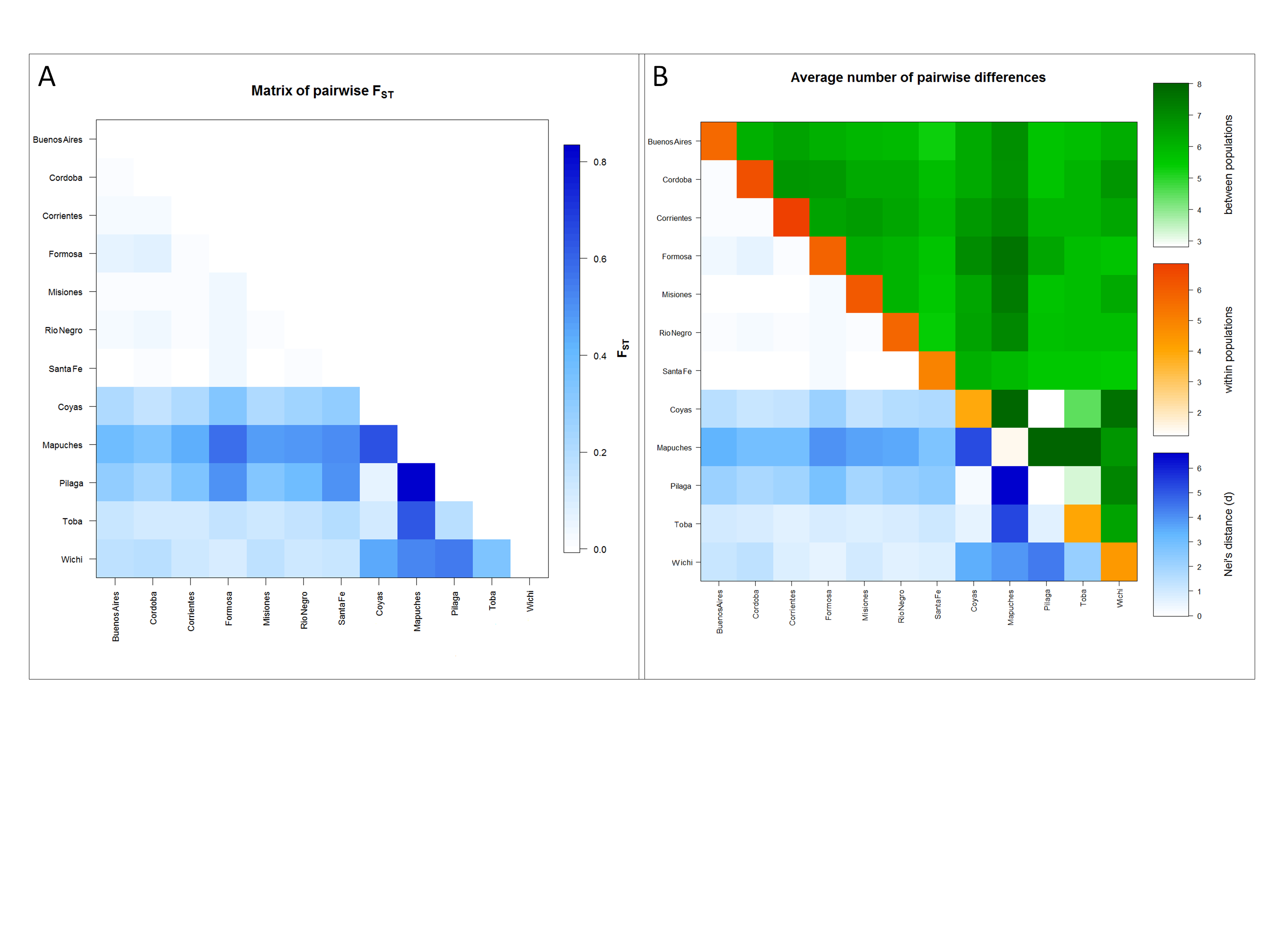

Supplement: Additional file 5 — Figure S3. Pairwise FST values (A), and average number of pairwise differences within and between populations and Nei's distances (B). [file 1471-2156-12-77-S5.TIFF]
